# Supplementary material for: Dysregulation of Immune Cell Subpopulations in Atypical Hemolytic Uremic Syndrome
Source: Int J Mol Sci. 2023 Jun 11;24(12):10007. doi: 10.3390/ijms241210007 (PMC10298405; doi:10.3390/ijms241210007)
Supplement: Supplementary file 1 [file ijms-24-10007-s001.zip › ijms-2423067-Supplementary Materials.pdf]

International Journal of *Molecular Sciences*

MDPI

Supplementary Materials

Dysregulation of Immune Cell Subpopulations in Atypical Hemolytic Uremic Syndrome

I-Ru Chen[1,2], Chiu-Ching Huang[1,2], Siang-Jyun Tu[3], Guei-Jane Wang[1,4], Ping-Chin Lai[2], Ya-Ting Lee[3,4,5], Ju-Chen Yen[3,4,5], Ya-Sian Chang[3,4,5] and Jan-Gowth Chang[1,3,4,5*]

[1] Graduate Institute of Clinical Medical Science, College of Medicine, China Medical University, No. 91 Hsueh-Shih Rd., North District, Taichung 40402, Taiwan
[2] Division of Nephrology and the Kidney Institute, Department of Internal Medicine, China Medical University and Hospitals, No. 2, Yude Rd., North Dist., Taichung 404332, Taiwan
[3] Center for Precision Medicine, China Medical University Hospital, No. 2, Yude Rd., North Dist., Taichung 404332, Taiwan
[4] Department of Medical Research, China Medical University Hospital, No. 2, Yude Rd., North Dist., Taichung 404332, Taiwan
[5] Epigenome Research Center, China Medical University Hospital, No. 2, Yude Rd., North Dist., Taichung 404332, Taiwan
* Correspondence: D6781@mail.cmuh.org.tw

Citation: Chen, I.-R.; Huang, C.-C.; Tu, S.-J.; Wang, G.-J.; Lai, P.-C.; Lee, Y.-T.; Yen, J.-C.; Chang, Y.-S.; Chang, J.-G. Dysregulation of Immune Cell Subpopulations in Atypical Hemolytic Uremic Syndrome. *Int. J. Mol. Sci.* **2023**, *24*, 10007. https://doi.org/10.3390/ijms241210007

Academic Editor: Jaap A. Joles

Received: 13 May 2023
Revised: 30 May 2023
Accepted: 7 June 2023
Published: 11 June 2023

Copyright: © 2023 by the author. Licensee MDPI, Basel, Switzerland. This article is an open access article distributed under the terms and conditions of the Creative Commons Attribution (CC BY) license (https://creativecommons.org/licenses/by/4.0/).

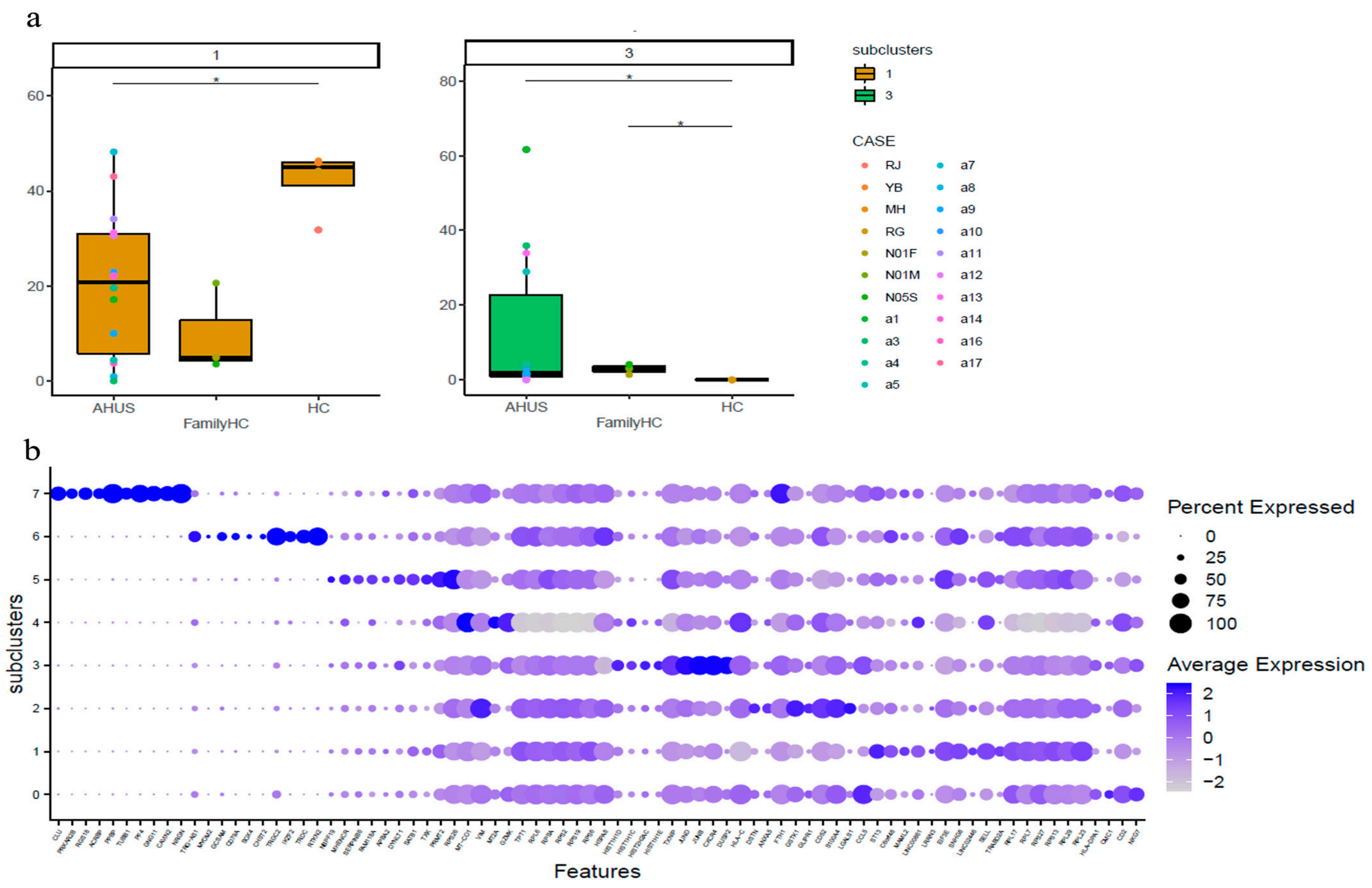

Figure S1. The figure presents boxplots displaying the subcluster significant abundance of central memory CD8 T-cells (a) in PBMCs of individuals with aHUS, aHUS family, and healthy subjects. (b) Dot plots of the gene expression profiles of the top 10 marker genes in each subcluster are also provided. Statistically significant differences are indicated by *P < 0.05 and **P < 0.01.

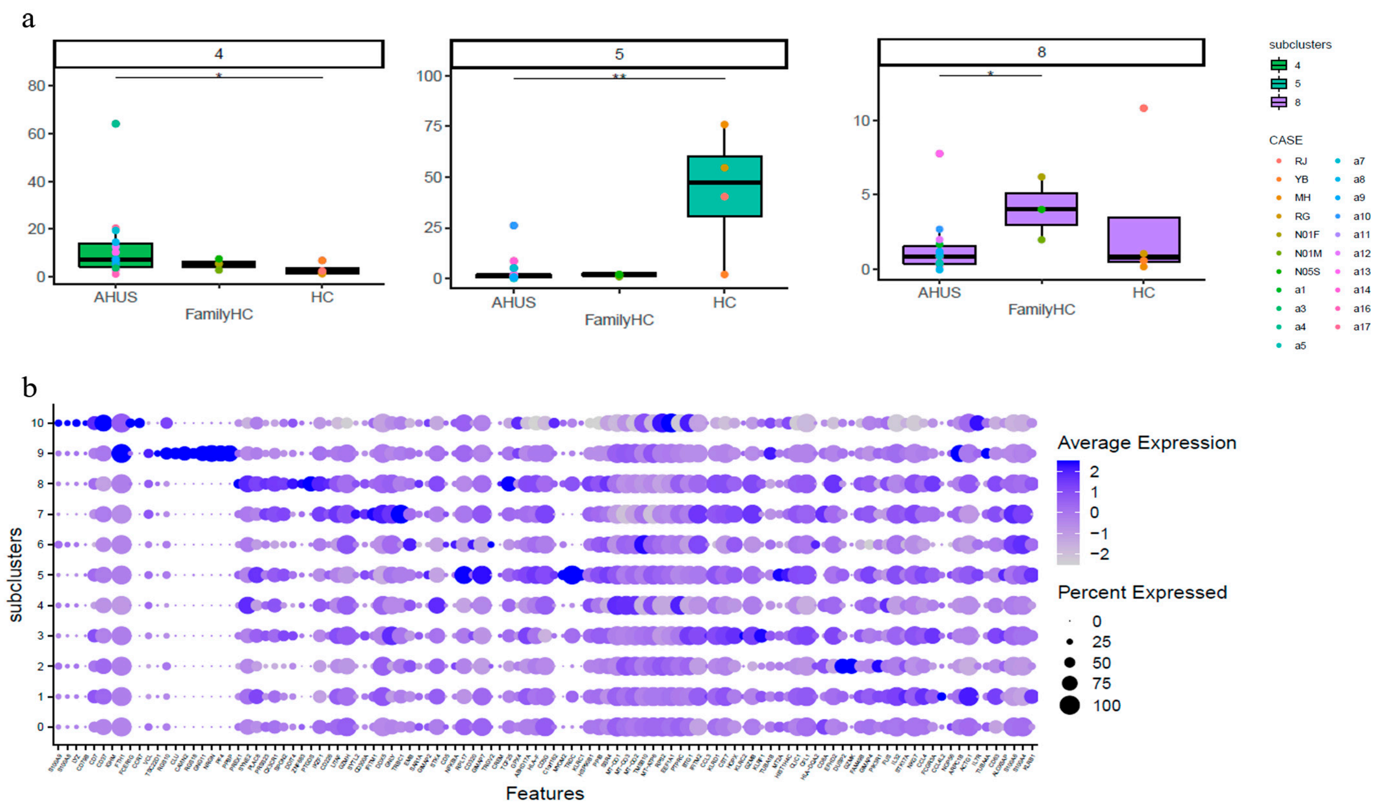

Figure S2. The figure presents boxplots displaying the subcluster significant abundance of non-Vd2 gd T-cells (a) in PBMCs of individuals with aHUS, aHUS family, and healthy subjects. (b) Dot plots of the gene expression profiles of the top 10 marker genes in each subcluster are also provided. Statistically significant differences are indicated by *P < 0.05 and **P < 0.01.

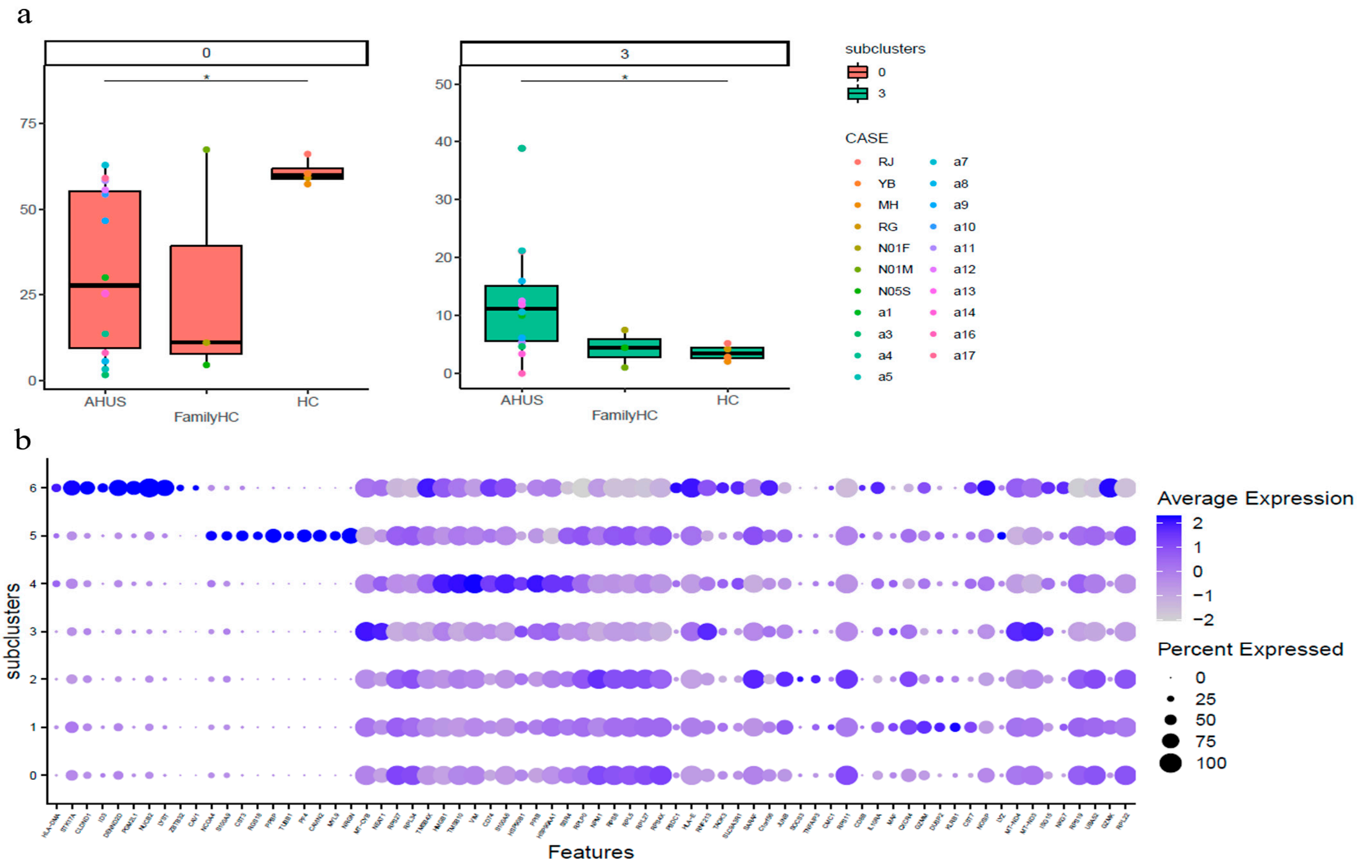

Figure S3. The figure presents boxplots displaying the subcluster significant abundance of Th1 cells (a) in PBMCs of individuals with aHUS, aHUS family, and healthy subjects. (b) Dot plots of the gene expression profiles of the top 10 marker genes in each subcluster are also provided. Statistically significant differences are indicated by *P < 0.05 and **P < 0.01.

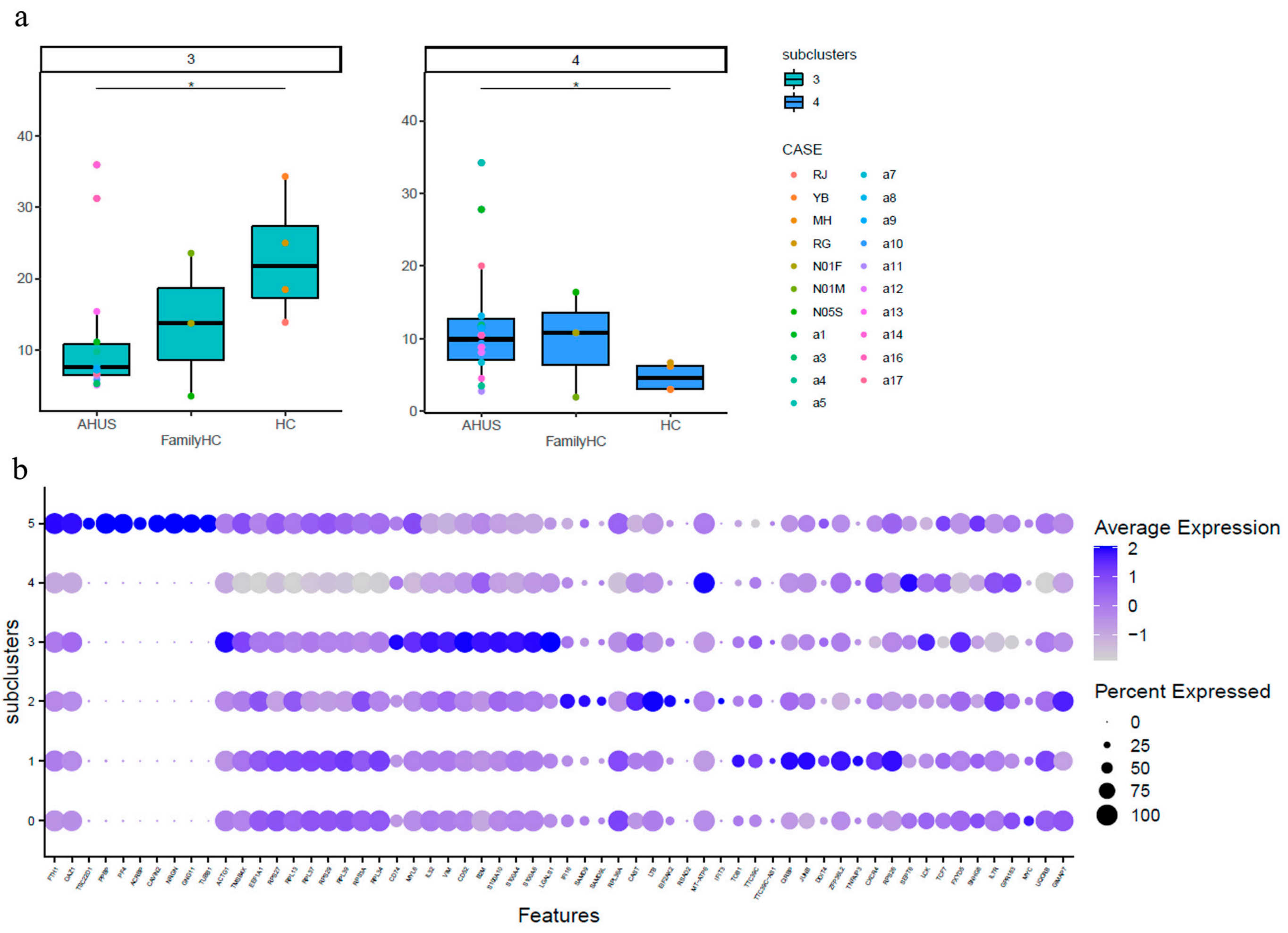

Figure S4. The figure presents boxplots displaying the subcluster significant abundance of Th17 cells (a) in PBMCs of individuals with aHUS, aHUS family, and healthy subjects. (b) Dot plots of the gene expression profiles of the top 10 marker genes in each subcluster are also provided. Statistically significant differences are indicated by *P < 0.05 and **P < 0.01.

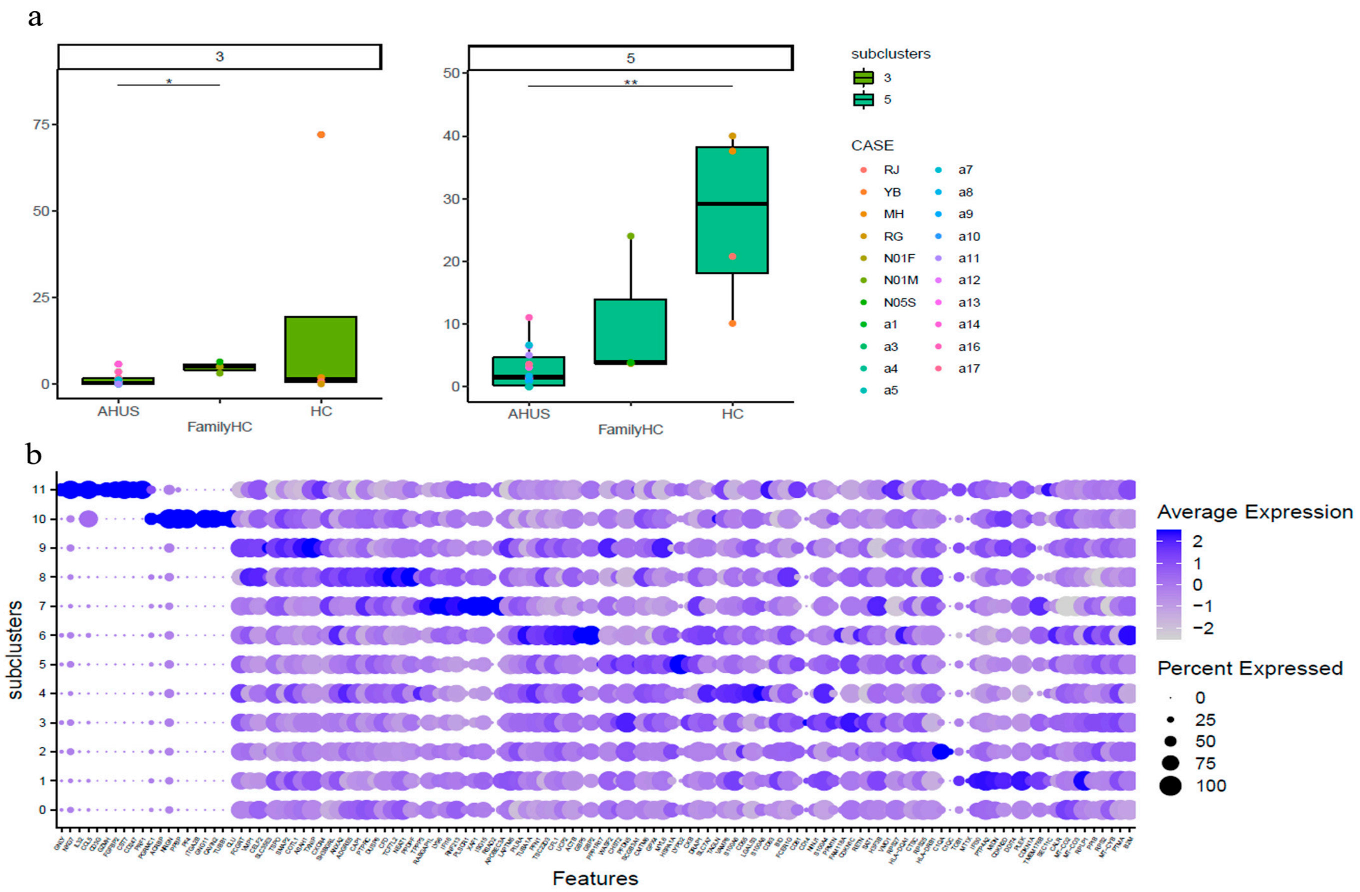

Figure S5. The figure presents boxplots displaying the subcluster significant abundance of non-classical monocytes (a) in PBMCs of individuals with aHUS, aHUS family, and healthy subjects. (b) Dot plots of the gene expression profiles of the top 10 marker genes in each subcluster are also provided. Statistically significant differences are indicated by *P < 0.05 and **P < 0.01.

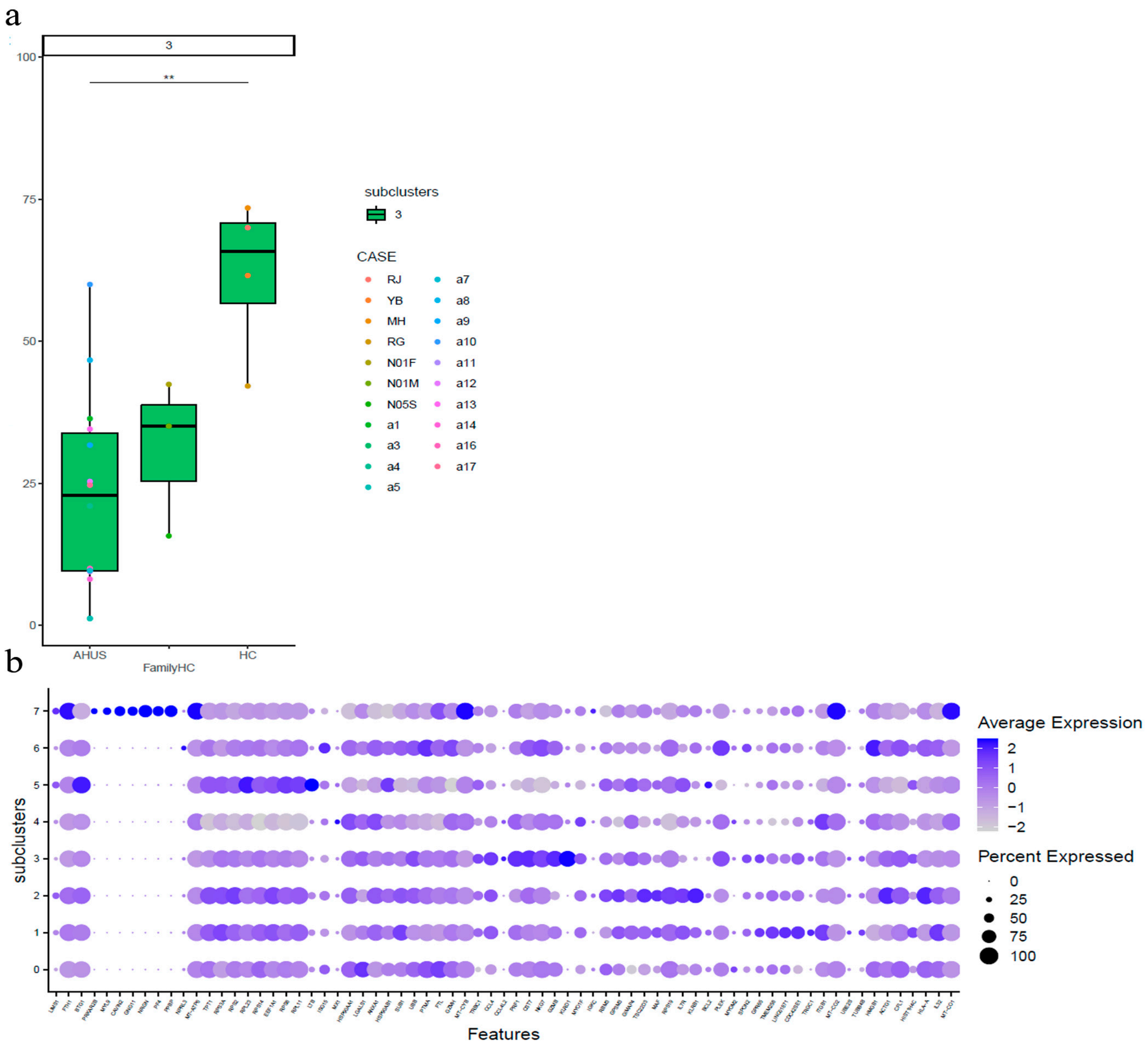

Figure S6. The figure presents boxplots displaying the subcluster significant abundance of terminal effector CD4-T cells (a) in PBMCs of individuals with aHUS, aHUS family, and healthy subjects. (b) Dot plots of the gene expression profiles of the top 10 marker genes in each subcluster are also provided. Statistically significant differences are indicated by *P < 0.05 and **P < 0.01.

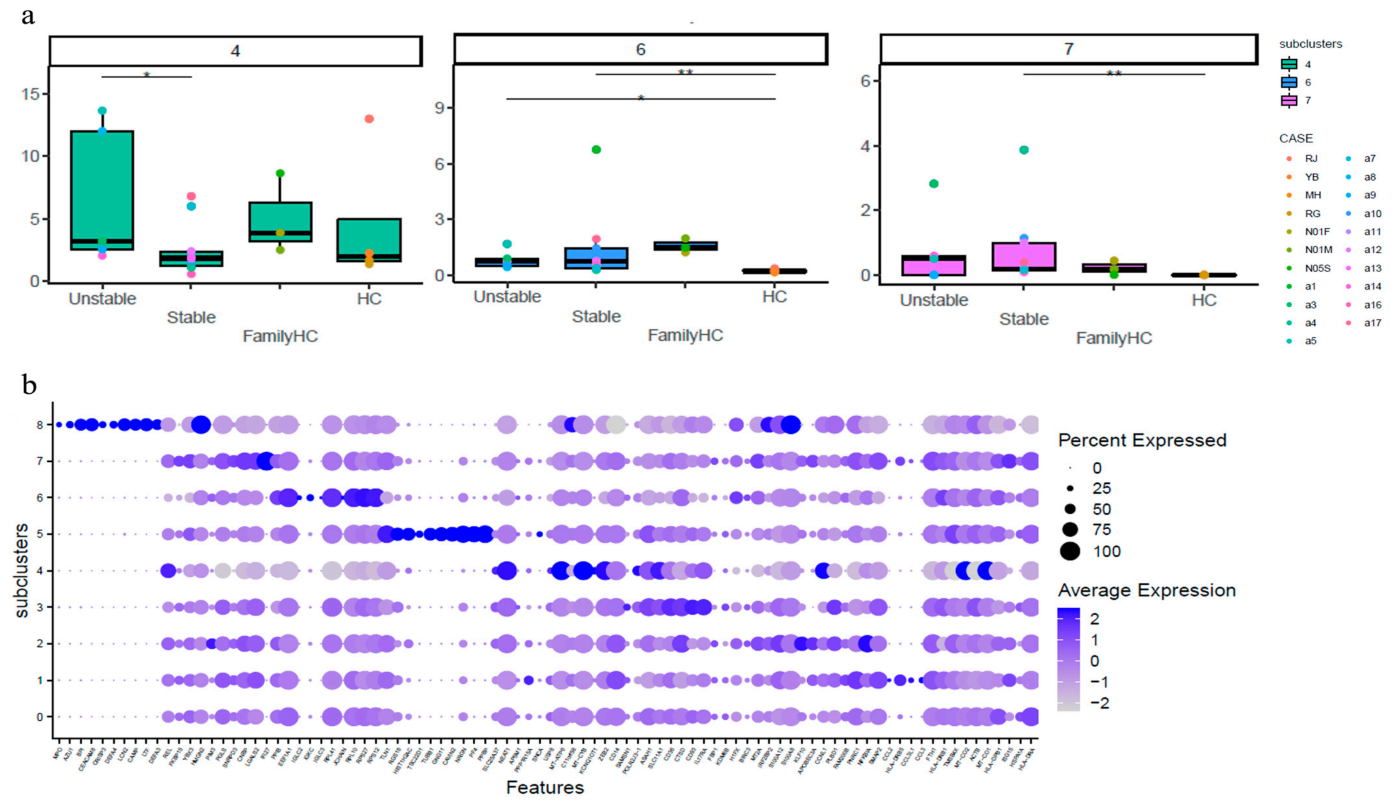

Figure S7. The figure (a) displays boxplots of subcluster significant abundance for classical monocytes in PBMCs of individuals with unstable and stable disease activity of aHUS, aHUS family, and healthy subjects, along with (b) dot plots of the gene expression profiles of the top 10 marker genes in each subcluster. Statistically significant differences are indicated by *P < 0.05 and **P < 0.01.

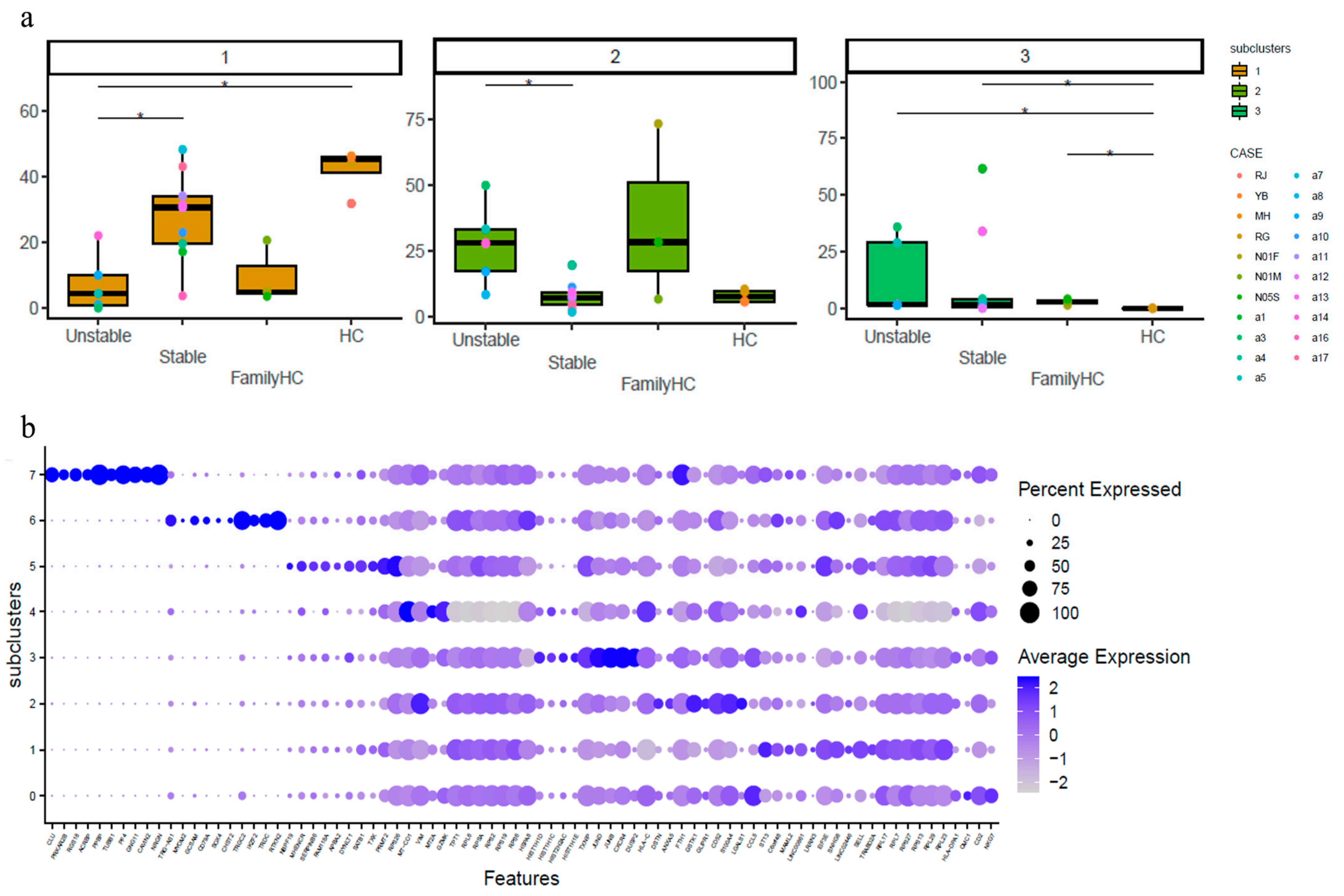

Figure S8. The figure (a) displays boxplots of subcluster significant abundance for boxplots of subcluster significant abundance for central memory CD8 T-cells in PBMCs of individuals with unstable and stable disease activity of aHUS, aHUS family, and healthy subjects, along with (b) dot plots of the gene expression profiles of the top 10 marker genes in each subcluster. Statistically significant differences are indicated by *P < 0.05 and **P < 0.01.

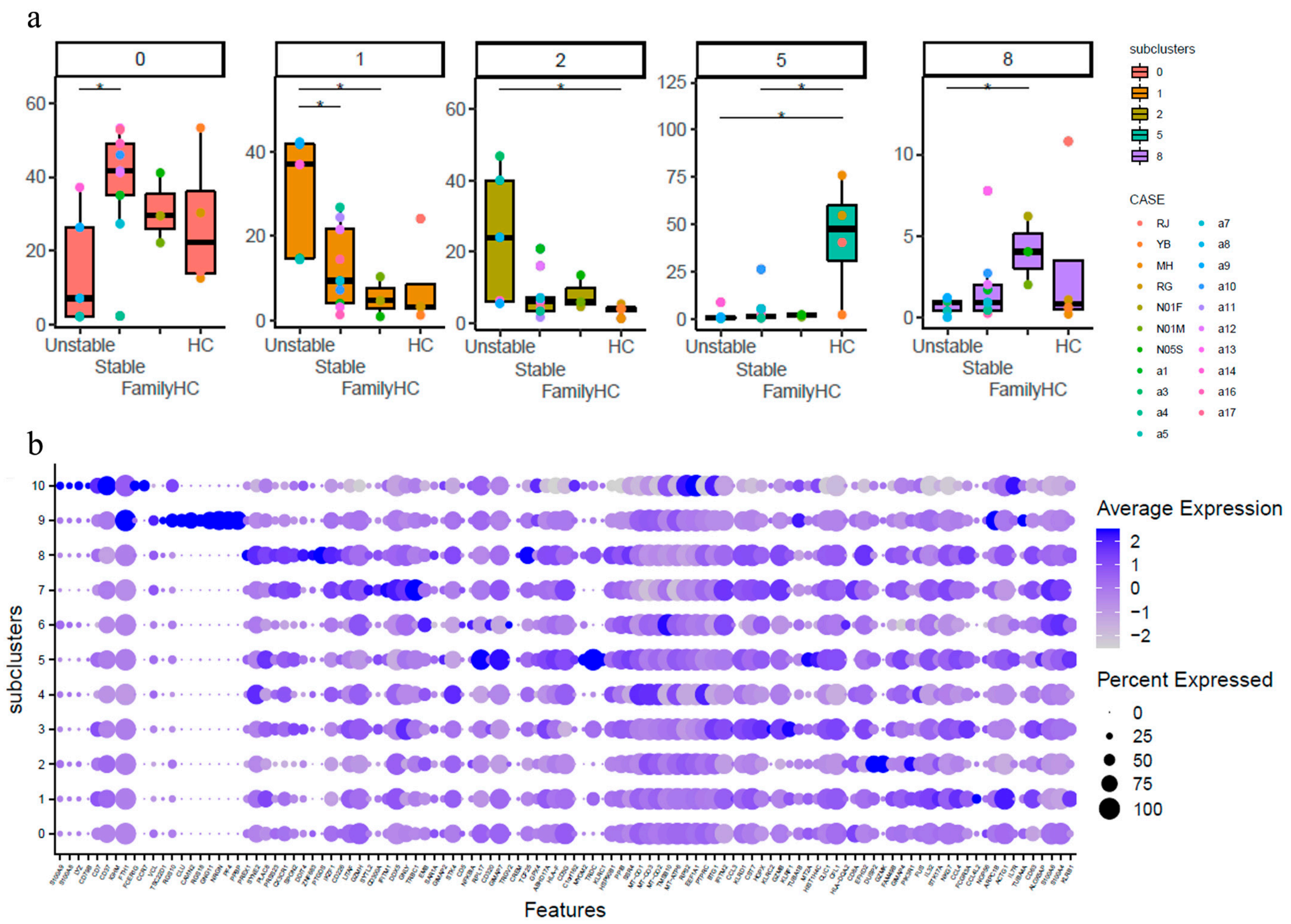

Figure S9. The figure (a) displays boxplots of subcluster significant abundance for boxplots of subcluster significant abundance for non-Vd2 gd T-cells in PBMCs of individuals with unstable and stable disease activity of aHUS, aHUS family, and healthy subjects, along with (b) dot plots of the gene expression profiles of the top 10 marker genes in each subcluster. Statistically significant differences are indicated by *P < 0.05 and **P < 0.01.

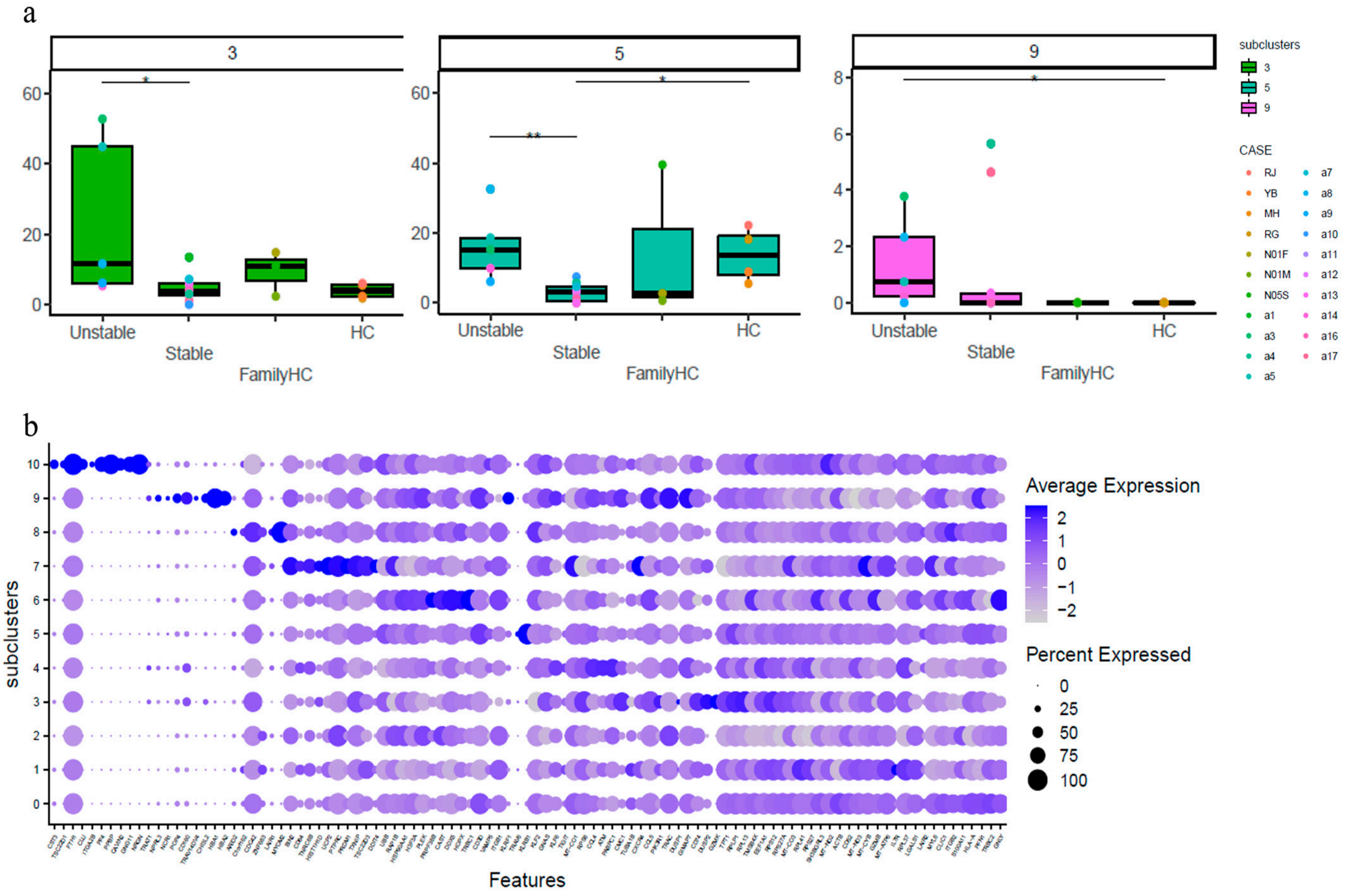

Figure S10. The figure (a) displays boxplots of subcluster significant abundance for boxplots of subcluster significant abundance for terminal effector CD8 T-cells in PBMCs of individuals with unstable and stable disease activity of aHUS, aHUS family, and healthy subjects, along with (b) dot plots of the gene expression profiles of the top 10 marker genes in each subcluster. Statistically significant differences are indicated by *P < 0.05 and **P < 0.01.

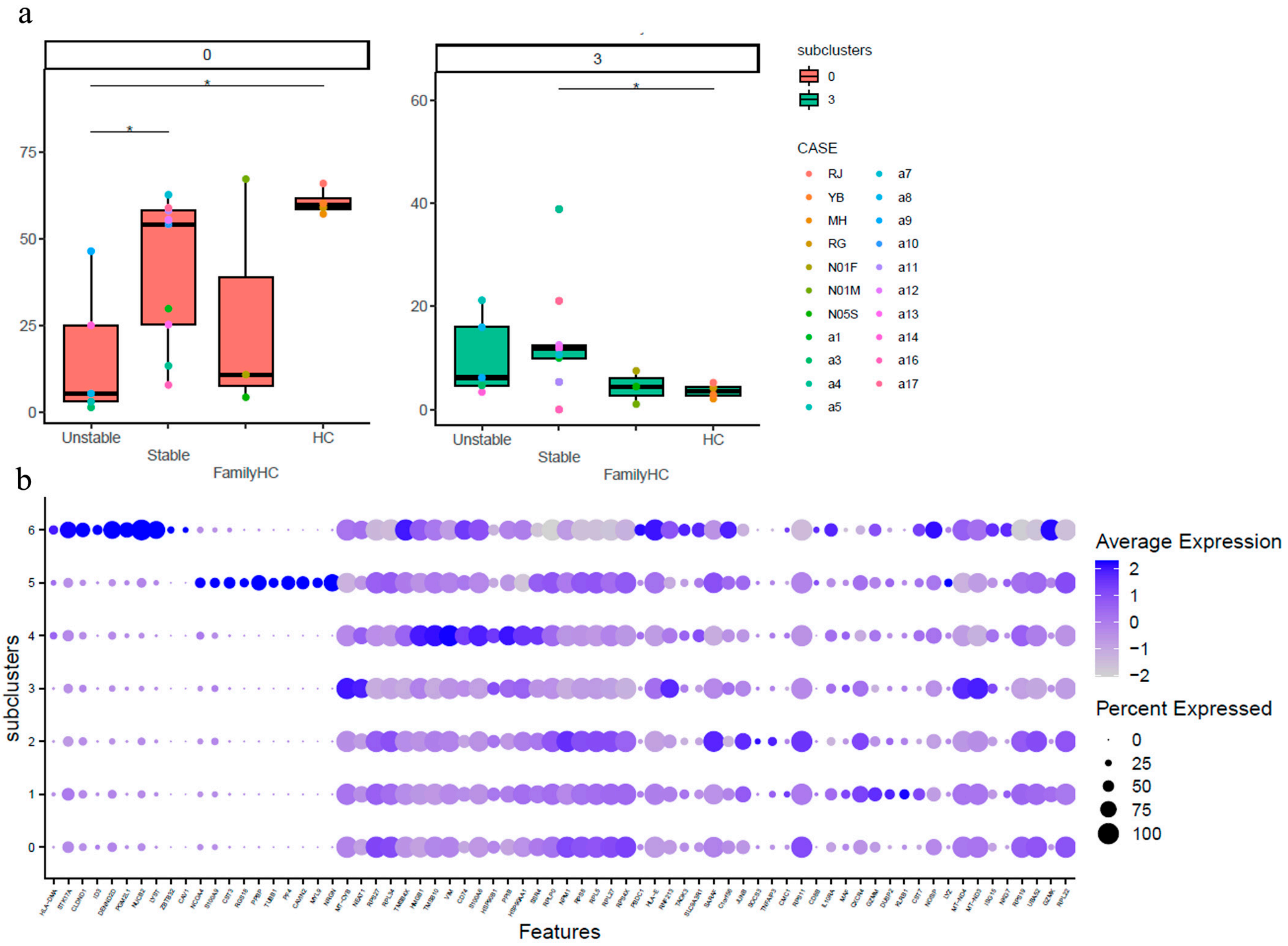

Figure S11. The figure (a) displays boxplots of subcluster significant abundance for boxplots of subcluster significant abundance for Th1 cells in PBMCs of individuals with unstable and stable disease activity of aHUS, aHUS family, and healthy subjects, along with (b) dot plots of the gene expression profiles of the top 10 marker genes in each subcluster. Statistically significant differences are indicated by *P < 0.05 and **P < 0.01.

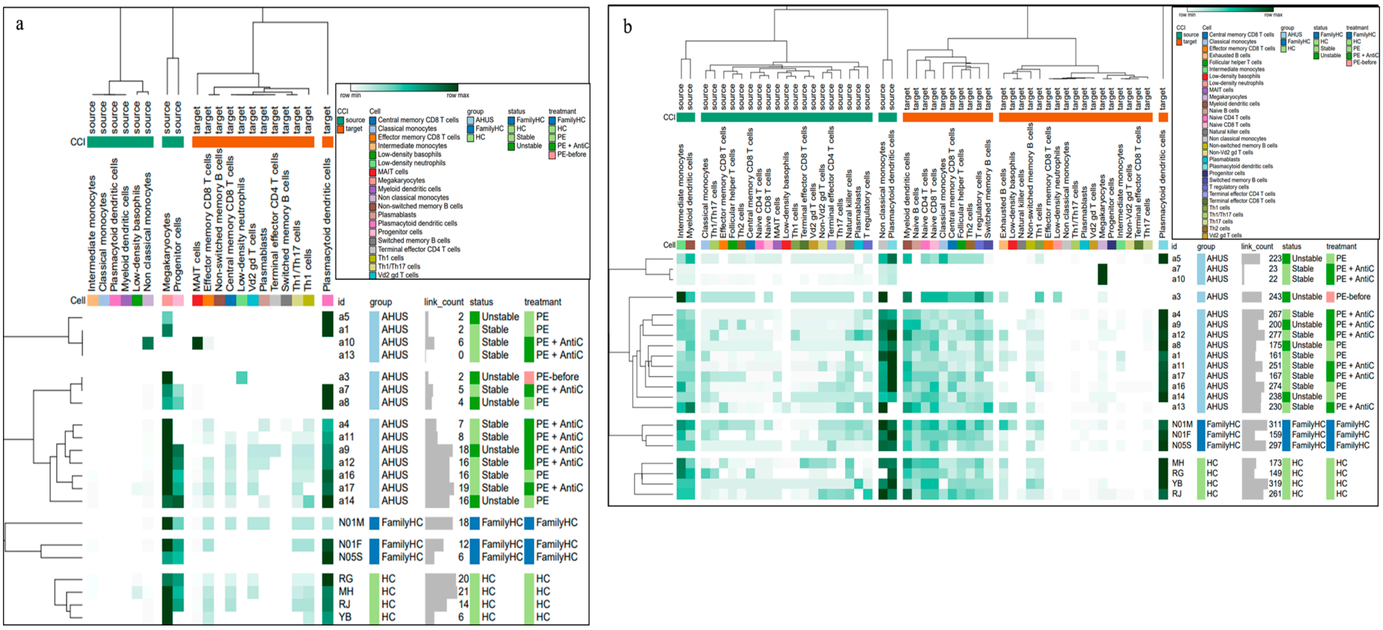

Figure s12. Cell-cell interaction signaling of CXC (a), and SELPLG (b) among individuals with aHUS with varying disease activity, treatment, aHUS family members, and healthy controls.

Disclaimer/Publisher's Note: The statements, opinions and data contained in all publications are solely those of the individual author(s) and contributor(s) and not of MDPI and/or the editor(s). MDPI and/or the editor(s) disclaim responsibility for any injury to people or property resulting from any ideas, methods, instructions or products referred to in the content.
